# Possible protective effects of vanillin against stress-induced seminiferous tubule injury via modulation of Nrf2 and ZO1

**DOI:** 10.1007/s00210-024-03355-6

**Published:** 2024-08-26

**Authors:** Sara Mohamed Naguib Abdel Hafez, Entesar Ali Saber, Neven Makram Aziz, Mohammed Mustafa Abd El Aleem, Manar Samir Mohamed, Elshimaa M. N. Abdelhafez, Randa Ahmed Ibrahim

**Affiliations:** 1 Department of Histology and Cell Biology, Faculty of Medicine, Minia University, Minia, Egypt; 2 Department of Medical Sciences (Histology and Cell Biology), Deraya University, New Minia City, Egypt; 3 Department of Medical Sciences (Physiology), Deraya University, New Minia City, Egypt; 4 Vice president and Head of Medical Department , Deraya University, New Minia City, Egypt; 5 Department of Internal Medicine, Deraya University, Minia, Egypt; 6 Department of Medicinal Chemistry, Faculty of Pharmacy, Minia University, Minia, Egypt

**Keywords:** Immobilization stress, Vanillin, ZO-1, CD34, TNF-α, Nrf2, Docking

## Abstract

Around 20% of the human population is distressed. Previous studies have looked into the relationship between restraint immobilization stress (IS) and sexual behavior in male rats. The current study aimed to provide a brief explanation of the mechanisms that generated testicular injury with chronic IS and an attempt to evaluate the mechanisms and effects of vanillin as a novel protective agent. Forty-eight adult male albino rats were divided into six groups: control, vanillin-treated, chronic 2-h IS, 2-h stressed-vanillin-treated, chronic 6-h IS, and 6-h stressed-vanillin treated. The rats were sacrificed, and blood samples were collected for biochemical study. The testes were processed for biochemical and histological study, as well as histological Johnsen score. The results showed that prolonged IS increased both corticosterone and TNF-α levels as well as decreased testosterone, luteinizing hormone, catalase, and Nrf2 levels. This effect was more pronounced after 6 h of IS compared to 2 h. It also induced various testicular injuries with weak ZO-1 and CD34 immunoreactions. On the contrary, vanillin improved all mentioned biochemical and histological alternations induced by stress. Additionally, computational molecular docking analyses were conducted on the compound vanillin within the active site of Zona Occludens-1 (PDB ID: 2JWE). The results demonstrated remarkable docking scores and binding affinity, corroborating its potential protective efficacy. It could be concluded that vanillin is a promising treatment alternative for protecting testicular tissue from the harmful effects of IS via its antioxidant and anti-inflammatory properties.

## Introduction

Stress induces physiological, histological, and behavioral alterations. These modifications enable the individual to adapt to stressful events (Zareian et al. 2015). However, persistent stress has a detrimental effect on tissues, limiting various biological activities and negatively altering cellular proliferation and differentiation (Tsigos et al. 2020).

Male reproductive health has deteriorated significantly during the last many decades. In particular, a global drop in sperm quality has increased infertility rates. Male infertility is caused by a combination of variables, including spermatogenesis failure, sperm transportation problems, genetic flaws, hormone dysfunction, aging, and environmental and lifestyle factors. Among these elements, physiological and psychological stress appears to be one of the main causes of male reproductive failure (Karna et al. 2020).

Several researchers have found that persistent stress causes morphological and functional changes in the testes. Rats exposed to chronic stressors demonstrated impaired sperm production and decreased blood testosterone levels (Ribeiro et al. 2018). Immobilization stress was associated with altered androgen biosynthesis, accompanied by higher levels of serum-luteinizing hormone and follicle-stimulating hormone that coincided with a reduction in testosterone (Karna et al. 2020).

The blood-testis barrier (BTB) is mainly made up of widespread tight junctions (TJs) between Sertoli cells. Tight junctions in Sertoli cell membranes form the structural foundation of the blood-testis barrier. The blood-testis barrier’s TJs are made up of two transmembrane proteins: claudin-11 and occludin. Zonula occludens-1 (ZO-1) is an adapter protein that connects transmembrane proteins with the actin cytoskeleton. It was found on the apical membrane of Sertoli cells in contact with mature spermatids in the BTB (Luaces et al. 2023).

Telocyte (Tc) is a novel type of classic interstitial cell identified in a variety of tissues, including the human testis. Telocytes are found in the peritubular region of the testis and communicate with myoid via cell junctions. They also communicate with blood vessels and androgen-producing interstitial cells (Leydig cells) via lengthy cytoplasmic projections (Liu et al. 2019). The Tc establishes homo- and intercellular junctions, vesicle release, and paracrine and/or autocrine signaling. They also connect and communicate with Leydig myo-epithelial cells, and blood vessels through lengthy cytoplasmic projections, being responsible for transporting substances between the interstitial and the seminiferous tubule, such as testosterone, which is required for spermatogenesis (Gomes et al. 2021).

Vanillin, which its chemical name is (4-hydroxy-3-methoxybenzaldehyde), is considered the primary component of vanilla. It is present in numerous essential plant oils (Liu et al. 2023). It is commonly utilized in processed foods, pharmaceuticals, and perfumes (Mohamed 2019). Previous research has established the anti-oxidant, anti-microbial, anti-inflammatory, anti-apoptotic, anti-cancer, neuro-protective, hepato-protective, reno-protective, and cardio-protective effects of vanillin (Mentese et al. 2022).

The previous docking studies highlight ZO-1’s multifaceted roles and its interactions with other proteins are stated as ZO-1 is a fascinating protein involved in cell junctions and plays critical roles in maintaining tissue integrity. A study published investigated the spatial organization of gap junctions and glutamate receptors in the outer plexiform layer (OPL) of the mammalian retina (Puller et al. 2009). ZO-1, known for its role in tight and adherents junctions, was closely associated with specific connexins (Cx) at gap junctions. In rabbit retinas, ZO-1 was found near Cx50 on dendrites of A-type horizontal cells. In mouse retinas, ZO-1 was associated with Cx36 at rod spherules and cone pedicle bases. The spatial arrangement of gap junctions and glutamate receptors suggested potential modulation of electrical synapses by glutamate release from photoreceptors. Another study explored the interaction between the cytoplasmic tail of the calcitonin receptor (CTR) and ZO-1 in tight junctions (Aljameeli et al. 2016). The PDZ-binding motif in the C-terminus of CTR was found to associate with the PDZ3 domain of ZO-1. Researchers developed an αCT1 peptide mimetic to competitively block interactions at the PDZ2 domain of ZO-1 (Obert et al. 2017). This approach aimed to inhibit ligands that selectively bind to ZO-1’s PDZ2 domain.

The purpose of this study was to investigate the potential effects of chronic immobilization stress on hypophysis-adrenal axis (HPA) activity by measuring corticosterone and testosterone hormone levels in order to better understand the fate of reproduction disability following HPA axis reactivity. This could serve as a foundation for future research into stress-induced male reproductive problems. Furthermore, despite vanillin’s stated medicinal characteristics, little is known about its therapeutic potential for testicular dysfunction. The current study sought to evaluate vanillin’s therapeutic impact against testicular damage caused by prolonged stress.

## Material and methods

### Experimental animals

#### Ethical approval

The Ethics Committee “FMREC” Faculty of Medicine, Minia University, Minia, Egypt, accepted this study based on the source of the rats, inclusion and exclusion criteria, caging, comfort, health state, and comprehensive experimental design and technique. Approval Number: 549/2022.

In this study, 48 apparently healthy adult male Wistar rats weighing between 150 and 200 gm of average age 8–10 weeks (pathogen-free) were employed. These were received from the Animal House, Faculty of Medicine, Minia University, and all animals were housed in a clean, air-conditioned room. Food and water were provided ad libitum. Two weeks of acclimatization were allowed at room temperature, with 12 h of darkness and 12 h of light. Rats were randomly placed into six groups of eight each. The six groups were housed in 12 stainless steel cages (four rats per cage), with each group separated into two cages to allow for unrestricted movement and roaming (40 cm by 40 cm by 25 cm).

### Immobilization stress method

Twenty-one days after the start of the experiment (duration of vanillin intake), stressed rats were lightly anaesthetized with ether for 40 ± 5 s and then immobilized (for 2 or 6 h) by attaching the four limbs of each animal in a prone position to a wooden board with adhesive zinc oxide hospital tape at room temperature (25 ± 2 °C). The head’s movement was controlled by keeping it in a metal loop coiled around the neck. To reduce pain and discomfort, the tape was unraveled after being moistened with acetone. In the unstressed group (control and vanillin-treated groups), the rats were kept in an animal cage in the experimental room (Aziz et al. 2013).

### Experimental design

#### Drug

Vanillin)4-hydroxy-3-methoxybenzaldehyde), with molecular weight 152.15 g/mol, was obtained from Sigma-Aldrich, CAS Number (121–33-5), 99% purity, Johannesburg, South Africa.

Rats were randomly assigned into six equal groups (eight rats) as follows: *control group (C)*, in which rats were fed on standard pellet chow. *Vanillin-treated group*, in which rats received daily oral water–dissolved vanillin at a dose 150 mg/kg for 21 days as reported by Salau et al. (2021). *Chronic 2-h immobilization stress (C 2 IS) group*, in which rats were immobilized 2 h once a day from 10 a.m. to 12 a.m. for 10 days and received no medication (Saber et al. 2019). *Two hours stressed-vanillin treated (Vanillin C 2 IS) group*, in which rats were treated orally with water-dissolved vanillin at the same previously mentioned dose for 21 days. The previously treated rats were subjected to immobilization stress for 2 h for the following 10 days associated with receiving the same daily oral dose of the vanillin (150 mg/kg) 1 h before immobilization stress. *Chronic 6-h immobilization stress (C 6 IS) group*, in eight which rats were immobilized 6 h once a day from 10 a.m. to 4 pm for 10 days and received no medication (Abdel-Fattah and El-Sayed, 2018). *Six hours stressed-vanillin treated (Vanillin C 6 IS) group*, in which rats were treated orally with water-dissolved vanillin at the same dose for 21 days. The previously treated rats were subjected to immobilization stress for 6 h for the following 10 days associated with receiving the same daily oral dose of the vanillin (200 mg/kg) 1 h before immobilization stress.

Five hours before scarification, the rats from all groups were slowly injected intravenously using the tail vein with charcoal macromolecules (0.5 mg) suspended in normal saline. The charcoal was used as a foreign antigen for detecting phagocytic cells (Saber et al. 2019).

### Biochemical analysis

At the end of the experimental period, the animals were sacrificed by using 1.9% inhaled diethyl ether (0.08 ml/l of container volume) (Aledani et al. 2020). After, an overnight fasting blood samples were collected from the jugular vein left at room temperature in a tube containing 0.5% heparin as the anticoagulant, then centrifuged in a cooling (Hettich) centrifuge at 3000 rpm for 15 min. The obtained clear plasma was stored at − 80 °C until used for (a) spectro-photo-fluro-metric measurement of corticosterone concentration according to the methods of Silber et al. (1958). (b) Testosterone and luteinizing hormone (LH) levels were measured using enzyme-linked immunosorbent assay (ELISA) kits following the instructions of the manufacturer’s instructions (Kamiya Biomedical Company).

### Analysis of testis homogenates

Right testes from each rat from each group were extracted and separated from the surrounding fat and connective tissue. All right testes were sectioned longitudinally, stored at − 80 °C, and homogenized in cold potassium phosphate buffer (0.05 M, pH 7.4). The ratio of tissue weight to homogenization buffer was 1:10. The testicular homogenates were centrifuged at 5000 rpm for 10 min at 4° C. The resultant supernatant was used to determine (a) catalase activities (anti-oxidative markers) using colorimetric assay kits according to the recommendations of the manufacturer (Bio-diagnostic, Egypt). (b) Tumor necrosis factor alpha (TNF-α) level (inflammatory marker) by using rat’s TNF-α ELISA kit (Lab Vision Corporation, USA) according to the manufacturer’s instructions. (c) Nuclear factor erythroid 2-related factor 2 (Nrf2) (anti-oxidative markers) concentration by using the Nrf2 ELISA kit (Novus Biologicals, USA) according to the manufacturer’s instructions.

### Histological and immune-histochemical examination

#### For light microscopic study

The left testis from all rats from each group was fixed in Blouin’s solution for 24 h, then fixed in 10% formol saline. Five micrometer-thick paraffin sections were prepared and stained with hematoxylin and eosin stains (for structural changes), and Masson trichrome (for collagen fibers) with a blue or light bluish-green color (Bancroft and Layton 2013). Congo red is an anionic dye capable of depositing itself in amyloid fibrils and amyloid structure of protein aggregates with a pink or light red color (Dapson 2018). Immune histochemical study was done according to the manufacturer’s instructions. The first primary antibody was anti CD 34 (a marker for telocytes and their relationship with the surrounding structures). It is a rabbit monoclonal, Catalog Number (EP373Y), used at concentration 1:2500 from Abcam company, USA. The second antibody was monoclonal rabbit antibodies for anti-zonula occludens-1 (ZO-1) to detect blood-testis barrier (Catalogue number. EPR19945–224, Abcam) used at concentration 1:1000. The negative control slide followed the same immunohistochemical study without adding primary antibodies. The positive control for ZO-1 antibody was the rat spleen, while the positive control slide for anti-CD34 was the rat kidney tissue (slides not included).

### Morphometrical study

The maturation of germ cells in the seminiferous epithelium was categorized using the Johnsen score. It assigns numbers ranging from 1 to 10 to a cross-section of each tubule based on the following parameters: 10 = complete spermatogenesis and perfect tubules; 9 = many spermatozoa with disorganized spermatogenesis; 8 = only a few spermatozoa present; 7 = no spermatozoa, but many spermatids present; 6 = only a few spermatids present; 5 = no spermatozoa or spermatids, but many spermatocytes present; 4 = only a few spermatocytes present; 3 = only spermatogonia present; 2 = no germ cells present, but Sertoli cells present; and 1 = no germ cells and no Sertoli cells present. The mean Johnsen score was detected by selecting ten successive seminiferous tubules from each slide from each rat (Akhtar et al. 2020). The mean score was then calculated by dividing the sum of scores by the number of tubules examined. The mean surface area fraction of CD34 and ZO-1 immunostained cells was also detected. Data was measured in ten high-power fields. Measurements were made by Image analysis software, Image J (http://rsbweb.nih.gov/ij/; NIH, Bethesda). This was done in the Histology and Cell Biology Department, Faculty of Medicine, Minia University. The image analyzer was first calibrated automatically to convert the measurement units (pixels) produced by the image analyzer program into actual micrometer units. Each field was enclosed inside the standard measuring frame, and then the positively reacting protein immunostained areas were masked with a blue binary color to be measured.

#### Image capture

Images were captured digitally with a digital camera (CX-31, Tokyo, Japan) mounted on an Olympus BX40 microscope.

### Statistical analysis

Data were represented as means ± standard errors of the mean (SEM). Statistical analysis was performed using Graphpad Prism 5 software and significant differences between groups were done by a one-way ANOVA followed by a Tukey-Kramar post hoc test for multiple comparisons with a value of *P* < 0.05 considered statistically significant.

### Docking study

Maestro software v13.4 (Schrödinger, LLC, NY, USA, 2022–4) was used for the docking studies.

### Ligand preparation

LigPrep, also known as ligand preparation, is an application that allows us to create tautomeric, stereochemical, and ionization variants from a basic 2D structure into a 3D structure. A precise 3D molecular model can be produced with the aid of LigPrep. LigPrep application’s key feature is energy minimization using OPLS4 force filed score (Desai et al. 2012) The ligand 4-hydroxy-3-methoxybenzaldehyde was drawn using chemdraw software version 22.2.0. Then it was converted from 2 to 3D design using LigPrep tool in Maestro v13.4 (Jayaprakash et al. 2022). The Schrödinger suite was used to minimize the energy score (Desai et al. 2012); the relevant results are displayed in Tables 3 and 4.

### Protein preparation

The protein with PDB id 2JWE was obtained from PDB (http://www.pdb.org). The protein preparation workflow of the Schrodinger suite was used to process the ProPrep. The protein preparation process utilized the OPLS4 force field. The H-bond and one of the chains were kept, but the water molecules, heteroatoms, and residues were removed score (Desai et al. 2012).

### Active site prediction and grid generation

The active sites of zonula occludens-1 protein were investigated using the SiteMap tool of Schrödinger-Maestro v13.4. With the aid of the module glide v9.7 (Schrödinger, LLC, NY, USA, 2022–4); the receptor-grid is created. Grid generation is a representation of the physical characteristics required to carry out the ligand-docking process, such as the receptor’s volume (more precisely, its active site). The produced receptor grid is employed in the ligand docking procedure’s comparative docking studies. With coordination of *X* − 1.13, *Y* − 0.79, and *Z* 2.64, docking at the center of the binding cavity-grid box was generated.

### Glide standard precision ligand docking

In this study, we employed the Glide module within Schrödinger-Maestro version 13.4 for flexible ligand docking. The primary objective was to obtain precise and detailed insights into ligand–protein interactions. To enhance the accuracy of our docking scores, we incorporated Epik state penalties. Concerning the docking protocol, we utilized the standard precision (SP) mode for ligand docking and the SP mode ensures a balance between computational efficiency and accuracy. The penalties for non-cis/trans amide bonds are to improve the reliability of our results; we imposed penalties specifically on non-cis/trans amide bonds and this step accounts for the conformational flexibility of the ligand. The Van der Waals scaling factor and partial charge cutoff for ligand atoms were set to 0.80 as well as the partial charge cutoff was chosen as 0.15 in order to let these parameters influence the ligand’s interactions with the protein. The Grid-based docking was generated based on the protein’s active site (specifically the zonula occludens-1 receptor, PDB ID: 2JWE), served as the docking target. The input and output files were adjusted as the impact minimization output file for the drug molecule served as the input for the docking process. The ligand was docked against the grid, resulting in structural adjustments within the active site. A text document containing glide scores (docking scores) was generated, whereas the ligand with the lowest glide score represents the best-docked position. Notably, our study revealed that 4-hydroxy-3-methoxybenzaldehyde exhibited favorable docking interactions with the protein (PDB ID: 2JWE) where Fig. 7a illustrates this interaction, as indicated by the G-score. In summary, our investigation demonstrates the successful application of the SP mode in Glide for ligand docking, emphasizing the importance of accurate scoring and parameter selection.

## Results

### Survival rate

No deaths were detected in any groups.

### Biochemical results

#### Plasma levels of corticosterone

The results showed that the vanillin-treated group had no significant influence on corticosterone concentration if compared to the control group. Chronic stress exposure, whether for 2 or 6 h, resulted in the greatest corticosterone concentration across all experimental groups. On the other hand, treatment of vanillin before and during chronic immobilization stress (CSI) resulted in a significant decrease in corticosterone concentration, with a greater decrease in the C 2 IS group than in the C 6 IS group. The vanillin C 2 IS group had a much lower corticosterone concentration but was still greater than the control group (Table 1).

**Table 1:** The mean plasma levels of testicular hormones and corticosterone in all studied experimental groups

Groups	Control	Vanillin treated	C 2 IS	Vanillin C 2IS	C 6 IS	Vanillin C 6IS
Parameters						
Corticosterone						
(µg/ml)	42.3 ± 1.9					
	43.5 ± 0.9	84.6 ± 3.8^ab^	24.6 ± 2.8^abc^	126.9 ± 5.7 ^ab^	64.7 ± 4.6 ^abcd^	
Testosterone						
(ng/ml)	6.3 ± 0.3	6.7 ± 0.2	3.15 ± 0.1^ab^	5.1 ± 0.3 ^abc^	2.1 ± 0.1^ab^	3.9 ± 0.2 ^abcd^
LH						
(ng/ml)	13.6 ± 0.7	13.9 ± 0.9	6.8 ± 0.3 ^ab^	10.8 ± 0.5 ^abc^	4.5 ± 0.1 ^ab^	8.9 ± 0.6 ^abcd^

Results represent the mean ± S.E

*C 2 IS* chronic 2-h immobilization stress, *C 6 IS* chronic 6-h immobilization stress, *LH* luteinizing hormone

^a^Significant difference from the control group

^b^Significant difference from the vanillin group

^c^Significant difference of treated from its corresponding stressed group

^d^Significant difference of vanillin C 6 IS from vanillin C 2 IS,* P* < 0.05

#### Plasma levels of testosterone and luteinizing hormone

The current study found that testosterone and LH concentrations in the vanillin-treated group were the same as those in the control group, but stressed non-treated groups had the lowest levels compared to all experimental groups. In contrast, the vanillin C 2 IS group had significantly greater testosterone and LH concentrations than the vanillin C 6 IS group, but both groups were still lower than the control group (Table 1).

#### Oxidative status and inflammatory markers

The study found that administering vanillin to the C 2 IS group reduced testicular TNF-α levels and increased catalase and Nrf2 levels compared to the vanillin C 6 IS group and non-treated stressed groups. However, the control and vanillin-treated groups remained insignificant. The study found that CIS exposure for 2 or 6 h resulted in the greatest testicular TNF-α levels and the lowest levels of catalase and Nrf2 relative to other experimental groups (Table 2).

**Table 2:** Testicular oxidative and inflammatory markers in the different experimental groups

Groups	Control	Vanillin treated	C 2 IS	Vanillin C 2 IS	C 6 IS	Vanillin C 6 IS
Parameters						
Anti-oxidative markers	Catalase					
(U/g						
tissue)	9.45 ± 0.2	9.87 ± 0.1	4.9 ± 0.2 ^ab^	7.8 ± 0.4 ^abc^	3.4 ± 0.1 ^ab^	6.5 ± 0.2 ^abcd^
	Nrf2					
(pg/mg tissue)	85.2 ± 1.8	83.6 ± 1.9	56.8 ± 1.6 ^ab^	79.2 ± 1.2 ^abc^	42.8 ± 1.7 ^ab^	65.2 ± 1.5 ^abcd^
Inflammatory marker						
	TNF-α					
(pg/mg tissue protein)	22.6 ± 1.07	21.8 ± 1.03	45.2 ± 1.2 ^ab^	35.6 ± 1.4 ^abc^	67.8 ± 1.5 ^ab^	50.9 ± 1.7 ^abcd^

Results represent the mean ± S.E

*C2IS* chronic 2-h immobilization stress, *C6IS* chronic 6-h immobilization stress, *Nrf2* nuclear factor erythroid 2-related factor 2, *TNF-α* tumor necrosis factor-alpha

^a^Significant difference from the control group

^b^Significant difference chronic group from the acute group

^c^Significant difference recovery group from its corresponding group; *P* < 0.05

### Histological results

#### Light microscopic study

**Fig. 1:**
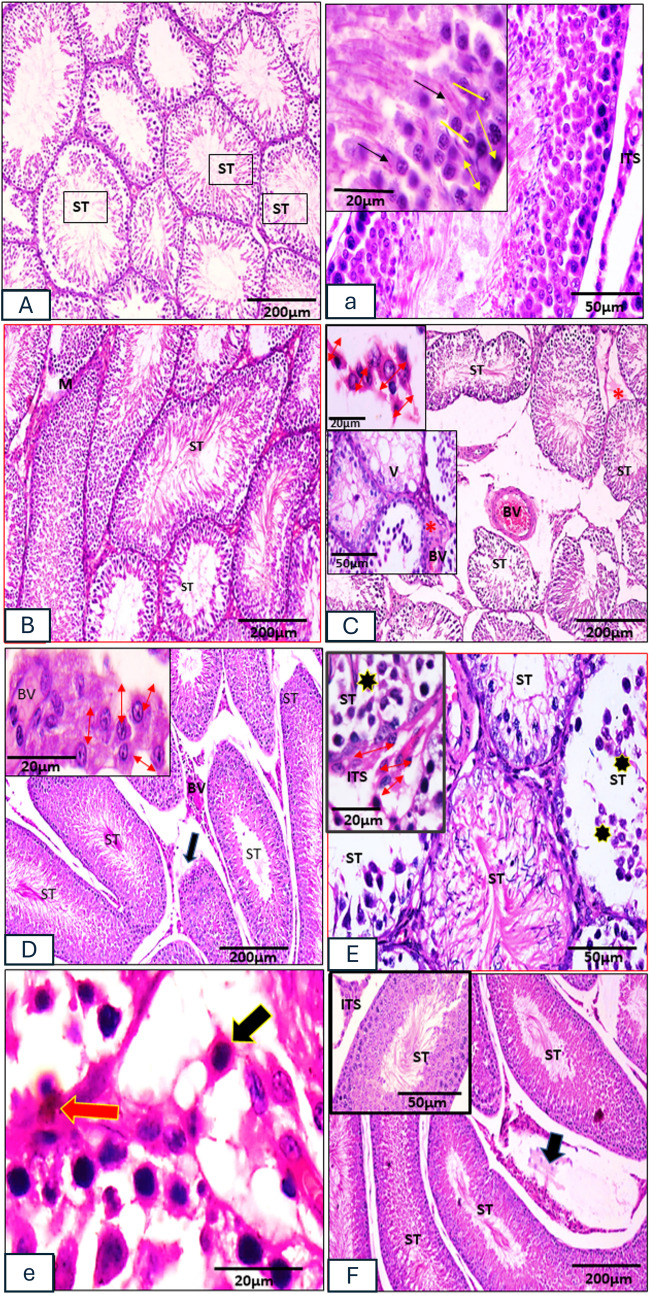
Photomicrographs of rat’s testicular tissue. (**A**, **a**) Control group. (**B**) Vanillin groups showing seminiferous tubules (ST) closely packed. (**a**) Seminiferous epithelium is formed of Sertoli cells (double head arrow) and layers of germ cells; spermatogenic cells (yellow arrow), primary spermatocytes (line), and spermatids (black arrows). Interstitial cells of Leydig (ITS) are also seen. (**C**) 2H IS group showing distorted and widely separated seminiferous tubules with depleted and vacuolated germinal epithelium (V). Notice congested and dilated blood vessels (BV) and acidophilic material (star) in the inter-tubular spaces containing large-sized macrophages with acidophilic cytoplasm (double arrows head). (**D**) Vanillin 2 H IS group showing apparent normal structural architecture, but some seminiferous tubules appear irregular and disrupted (black arrow) and some blood vessels seen dilated (BV). Polygonal acidophilic interstitial cells of Leydig (double arrows head) are noticed. (**E**) 6H IS group showing disorganized and massively degenerated seminiferous tubules with apoptotic spermatogenic cells, detached germ cells seen in the tubular lumens (stars) and apoptotic interstitial cells of Leydig (double arrows head). (**e**) Inter-tubular spaces containing large sized macrophages with acidophilic cytoplasm (black arrow). Notice the appearance of charcoal labeled macrophage (red arrow). (**F**) Vanillin 6H IS group showing most seminiferous tubules appear normal (ST). Notice few disturbed tubules (black arrow). (HX&E, A, B, C, D, F) × 100; scale bar = 200 µm. Insets in C and F × 400, scale bar = 50 µm. Insets in a C, E, e, D × 1000, scale bar = 20 µm

In H&E sections of *control and vanillin-treated groups*, the testicular tissue showed similar histological picture. They showed normal histological appearance; seminiferous tubules (STs) appeared organized and closely packed. Each tubule was surrounded by basement membrane enclosing myoid and lined with seminiferous epithelium. The seminiferous epithelium was formed of Sertoli and layers of germ cells in different stages of growth: spermatogonia, primary and secondary spermatocytes, spermatids, and spermatozoa. Spermatogonia were rested on the basement membrane characterized by dark nuclei, next to it primary spermatocytes that were the largest spermatogenic cells having large, heterochromatic nuclei, and occasionally mitotic figures could be seen in them. Spermatids and spermatozoa were seen in the tubular lumen. Sertoli cells were tall columnar cells, which span from the basement membrane to the lumen. Between the tubules, there was interstitial tissue containing Leydig cells that appeared large polygonal cells with acidophilic cytoplasm. Sparse connective tissue with interstitial cells of Leydig, which showed as big polygonal cells with vesicular nuclei, prominent nucleoli, and acidophilic cytoplasm, was present in the interstitial tissue between the STs (Fig. 1(A, a)). It was noticed in H&E sections of the *C 2 IS group*, shrinkage, distortion, and wide separation of seminiferous tubules with depleted and vacuolated germinal epithelium, as well as apoptotic cells among the basal spermatogonia and also Sertoli cells. There were many congested and dilated blood vessels besides acidophilic material in the inter-tubular spaces (Fig. 1(C)). Furthermore, H&E sections of the *vanillin C 2 IS group* revealed marked improvement in the structural architecture of the testis, except for alterations of some tubules. The alterations were in the form of irregularly disrupted tubules with reduced thickness of the germinal epithelium and dilatation of many interstitial blood vessels. The interstitial cells of Leydig appeared to have the normal polygonal shape and acidophilic cytoplasm (Fig. 1(D)). H&E sections of the *chronic 6 IS group* showed massively degenerated seminiferous tubules with apoptotic spermatogenic cells, thickened basement membranes that were separated at certain sites. There were many congested blood vessels, apoptotic interstitial cells of Leydig, and acidophilic material in the interstitial spaces between tubules. Moreover, detached germ cells were frequently seen in the tubular lumens (Fig. 1(E)). The inter-tubular spaces contain large sized macrophages with acidophilic cytoplasm. H&E sections of the *vanillin C 6 IS group* displayed seminiferous tubules with apparent increased spermatogenic cell layers. However, the tubules revealed variable sizes. There were no differences in the histological findings between the control and vanillin-treated groups except for some tubules appeared disrupted besides presence of acidophilic material and dilated blood vessels in the interstitial spaces. The basement membranes appeared thin (Fig. 1(F)).

**Fig. 2:**
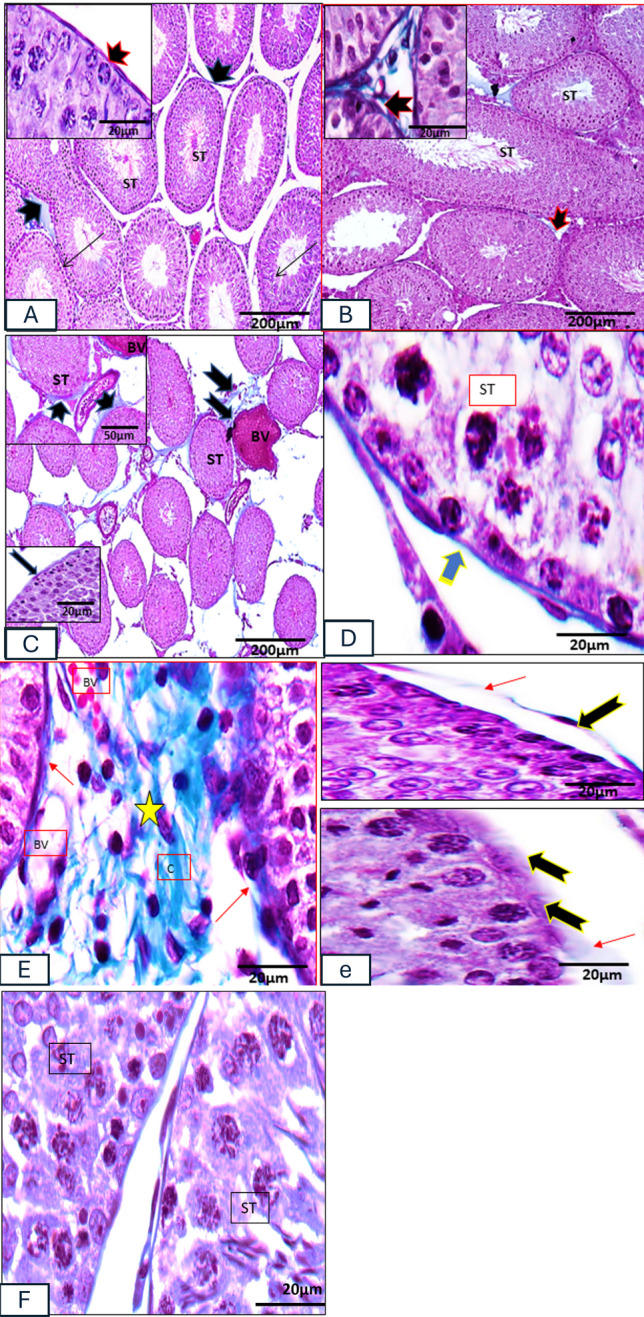
Photomicrographs of rat’s testicular tissue. (**A**, **B**) Control and vanillin groups respectively showing very minimal amount of collagen fibers in the interstitial tissue between seminiferous tubules (notched arrows), the inset showing normal basement membrane (arrow). (**C**) 2 H IS group showing apparent increase in the amount of collagen fibers (notched arrows) in the interstitial and perivascular spaces (BV). The lower inset showing thickened basement membrane. (**D**) Vanillin 2 H IS group showing the basement membrane appears with normal thickness and enclosing myo-epithelial cells, (stripped arrow). (**E**, **e**) 6H IS group showing extensive fibrosis (stars) of compact collagen bundles (**C**) in the interstitial tissues and around the blood vessels (BV). Notice the thickened basement membranes that may be separated at certain sites enclosing myoid (arrows). (F) Vanillin 6H IS group showing apparent less amount of collagen fibers and the basement membrane appear thin and enclosing myoid (stripped arrow). Masson’s trichrome stain: A, B, C × 100; scale bar = 200 µm. Insets C × 400; scale bar = 50 µm. Insets in A, B, C and D, E, e, F × 1000; scale bar = 20 µm

*Masson’s trichrome stain* of *control and vanillin-treated groups* showed a very minimal amount of collagen fibers in the interstitial tissue between seminiferous tubules (Fig. 2(A, B)). *Masson’s trichrome stain* of the *C 2 IS group* showed an apparent increase in the amount of collagen fibers in the interstitial tissue and around the blood vessels (Fig. 2(C)). *Masson trichrome stain* of the *vanillin C 2 IS group* showed an apparent reduction of fibrosis if compared with the stressed groups and the basement membrane appear with apparent normal thickness and enclosing myoid (Fig. 2(D)). *Masson’s trichrome stain* of *chronic 6 IS group* showed extensive fibrosis in the interstitial tissue and around the blood vessels. Thickened basement membranes that may be separated at certain sites enclosing myoid were noticed (Fig. 2(E)). *Masson’s trichrome stain* of the *vanillin C 6 IS group* showed less amount of collagen fibers if compared with the stressed groups (Fig. 2(F)).

**Fig. 3:**
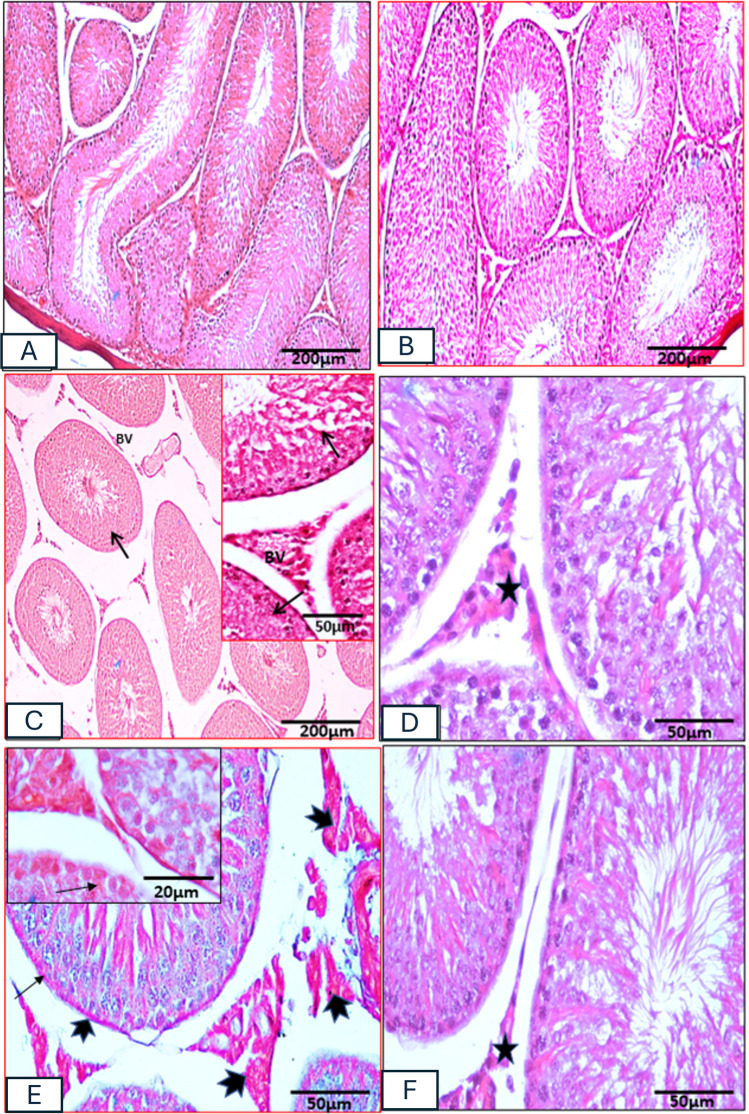
Photomicrographs of rat’s testicular tissue. (**a**, **b**) Control and vanillin groups respectively no amyloid deposition in the inter-tubular spaces. (**C**) 2 H IS group showing amyloid deposition within the interstitial space (stars), accumulate within the vascular walls and within the peritubular myoid (arrows). (**D**) Vanillin 2 H IS group showing unnoticeable amyloid deposition in the interstitial spaces (star). (**E**) 6H IS group showing extensive accumulation of amyloid in the interstitial spaces (notched arrows) and the parenchyma is affected (arrow). (F) Vanillin 6H IS group showing very minimal amyloid deposition in the interstitial spaces (star). Congo red stain A, B, C 100 × ; scale bar = 200 µm. D, E, F × 400; scale bar = 50 µm. Insets in C, E × 1000; scale bar = 20 µm

*Congo red stain* of the *control and vanillin-treated groups* showed no amyloid deposition in the inter-tubular spaces (Fig. 3(A, B)). *Congo red stain* of the *C 2 IS group* demonstrated extensive amyloid deposition with a pink or light red color. The amyloid may be deposited loosely within the interstitial space or accumulate within the vascular walls or within the peri-tubular myo-epithelial cell layer (Fig. 3(C)). Amyloid deposition in the interstitial spaces was hard to be noticed in the *vanillin C 2 IS group* (Fig. 3(D)). The *chronic 6 IS group* showed extensive accumulation of amyloid with secondary tubular degeneration and necrosis. Parenchyma was affected and the remnants of atrophic tubules are widely separated by clumps of amyloid (Fig. 3(E)). The *vanillin C 6 IS group* showed minimal accumulation of amyloid in the interstitial spaces (Fig. 3(F)).

### Immune histochemical Stain

#### Immunohistochemical staining of anti-Zonula Occludes’ antibody

**Fig. 4:**
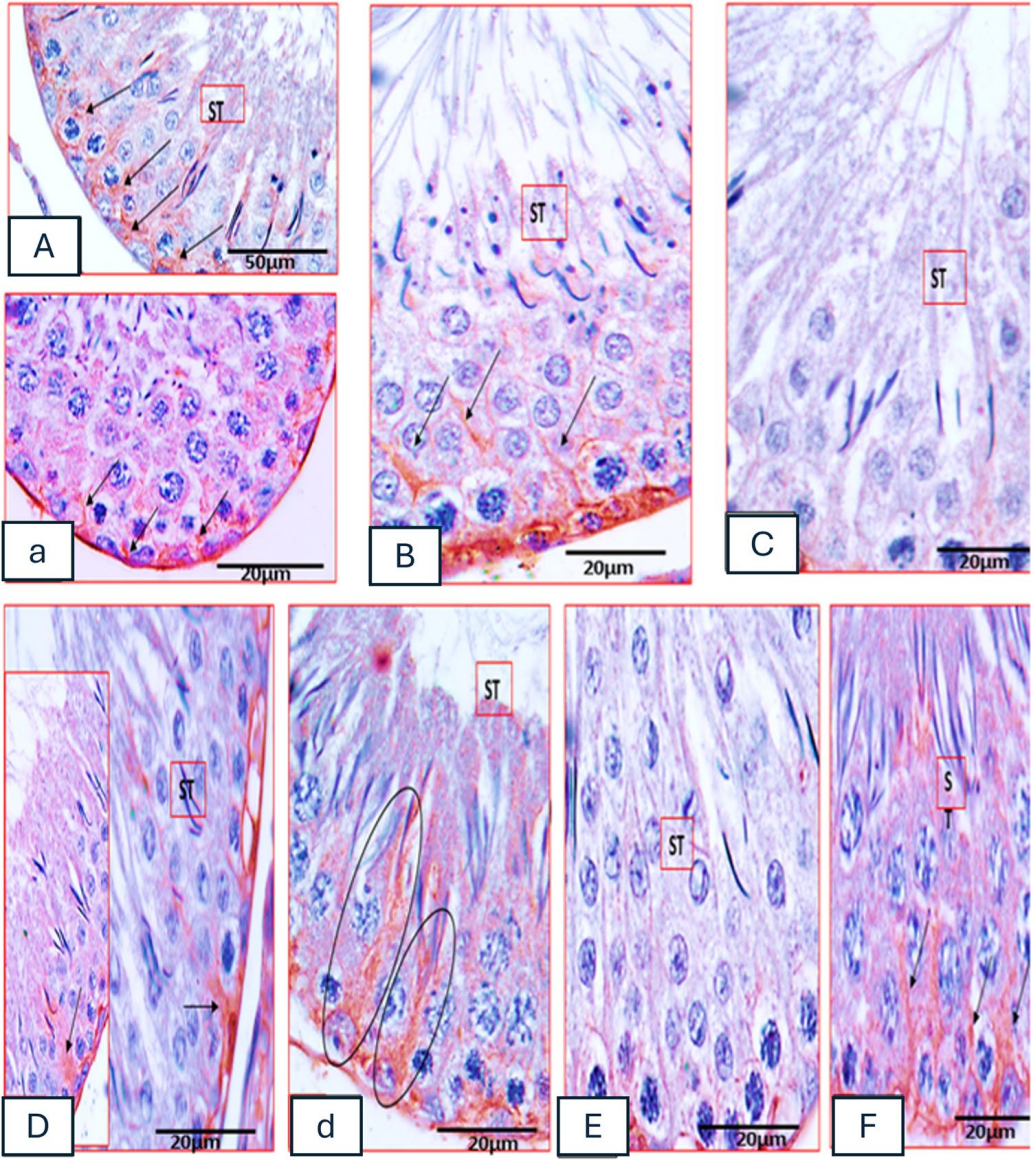
Photomicrographs of rat testicular tissue immune-stained with anti-zonula occludes antibodies. (**A**, **a**) Control group; ZO-immune-staining is strong transmembrane and localized between Sertoli cells at the basal compartment, consistent with the site of the blood-testis barrier (arrows). (**B**) Vanillin group; ZO-immune-staining localized between Sertoli cells at the basal compartment (arrows). (**C**) 2H IS group; ZO-immune-staining appears weak and diffuse. (**D**, **d**) vanillin 2H SI group; showing diffuse ZO-immune-staining around the base-lateral site, but also spread to stain the lateral site of Sertoli cells up to the ad-luminal compartment, as well as their cytoplasm (arrows and oval rings). (**E**) 6H IS group; ZO-immune-staining appears weak and diffuse. (**F**) vanillin 6H IS group showing weaker and more diffuse ZO-immune-staining around the base-lateral site, and spread to stain the lateral site of Sertoli cells up to the ad-luminal compartment, as well as their cytoplasm (arrows). Zonula Occludes “ZO” immune-staining × 1000 (Plus insets); scale bar = 20 µm except A × 400; scale bar = 50 µm

Both *control and vanillin groups* showed transmembrane immunoreactive ZO-1 reaction that restricted between Sertoli cells at the basal compartment, consistent with the site of the blood-testis barrier (Fig. 4(A)). Meanwhile, the *2 IS group* showed seminiferous tubular cells and ZO-1 immunostaining ZO-immune-staining appeared weak and diffuse (Fig. 4(C)). It was noticed in the *Vanillin C 2 IS group* the ZO-1 immunostaining became less than that of the control and vanillin-treated groups. Immune-staining was not only diffused around the base-lateral site, but also spread to stain the lateral site of Sertoli cells up to the ad-luminal compartment, as well as their cytoplasm (Fig. 4(D, d)). The *C 6 IS group* exhibited ZO-1 immune-staining which appeared weak and diffuse (Fig. 4(E)). The *Vanillin C 6 IS group* displayed that ZO-1 immunostaining became even weaker and more diffuse around the basolateral site but spread to stain the lateral site of Sertoli cells up to the ad-luminal compartment, as well as their cytoplasm (Fig. 4(F)).

#### Immune histochemical staining of anti-CD34 antibody

**Fig. 5:**
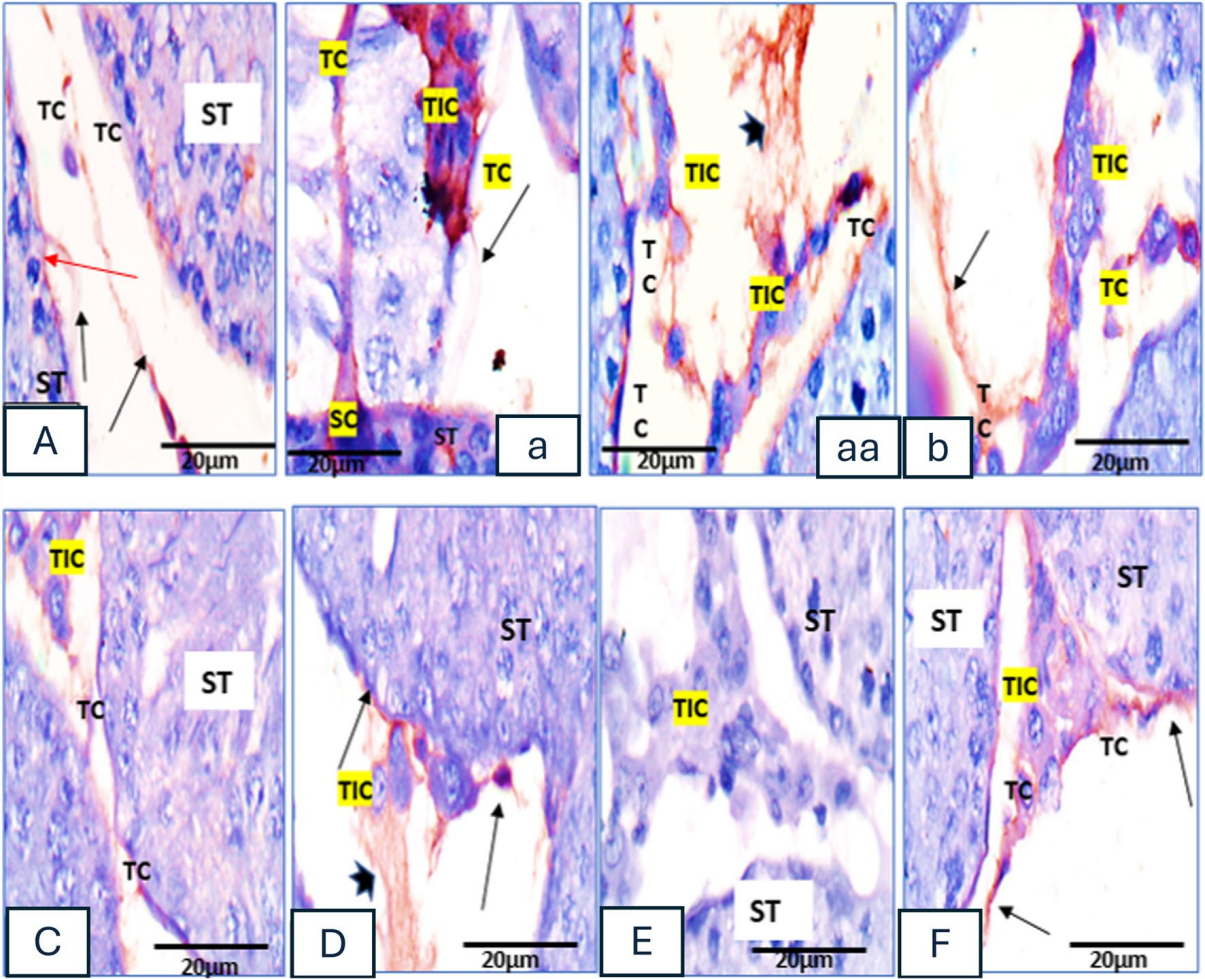
Photomicrographs of rat testicular tissue immune-stained with anti-CD34 antibodies. (**A**, a, aa) Control group showing strong CD34-immune-staining localized to the basement membrane of seminiferous tubules, in close contact myoid (red arrows). Telocytes appeared as small cell bodies with densely stained nuclei (TC) and very long processes (black arrows). Many telocytes (TC) and a dilated foot-like fragment (tailed arrow) around a Sertoli cell (SC). Many telocytes and a labyrinth structure (striped arrow) formed of intertwine telopodes with one another and in-between Leydig cells (TIC). (**B**) Vanillin group showing strong CD34-immune-staining of the long cytoplasmic processes of telocytes (TC) constituting a three-dimensional network around the interstitial cells (arrows). (**C**) 2H IS group; telocytes (TC) and the interstitial tissue (TIC) showing very weak CD34-immune-staining. (**E**) 6H IS group; telocytes (TC) and the interstitial tissue (TIC) showing negative CD34-immune-staining. (**D**–**F**) vanillin 2H&6H IS groups; expression of telocyte-marker protein CD34 in vanillin-treated groups is very near to that of the control figures (arrows) but more prominent in vanillin 2H IS group (CD34- immune-staining × 1000 scale bar = 20 µm)

Both control and vanillin groups showed that the expression of telocyte protein-marker “CD34” was localized to the basement membrane of seminiferous tubules, where they were in close contact with Sertoli cells and adjacent to peritubular myo-epithelial cells. Telocytes appeared as small cell bodies with densely stained nuclei and very long processes “telopodes.” The long cytoplasmic processes had thin segments and dilated regions constituting a three-dimensional network mainly around the interstitial cells (Fig. 5(A, a)). Dilated fragments of the telopodes form foot-like structures mainly around Sertoli cells (Fig. 5(a)). In some cases, the telopodes intertwine with one another and in-between adjacent Leydig cells and form characteristic labyrinths (Fig. 5(aa)). The C 2 IS group showed that the reaction of telocyte-marker protein CD34 was very weak (Fig. 5(C)), while in the C 6 IS group, the reaction of telocyte-marker protein CD34 was very faint/negative (Fig. 5(D)).

##### Vanillin-treated C 2IS and C 6IS groups

The level of telocyte-marker protein CD34 in vanillin-treated groups was very close to that of the control group but the intensity of the reaction was more pronounced in vanillin-treated stressed group for 2 h more than that of vanillin-treated stressed group for 6 h (Figs. 5(E) and 4(F)).

### Morphometrical result

Johnsen scoring and the mean surface area fraction of anti ZO-1 and CD 34 were significantly lower in groups C 2 IS and C 6 IS compared to groups control and vanillin, while these parameters were significantly higher in vanillin-treated C 2IS and C 6IS groups if compared with their corresponding stressed groups (Histogram I).

**Histogram I:**
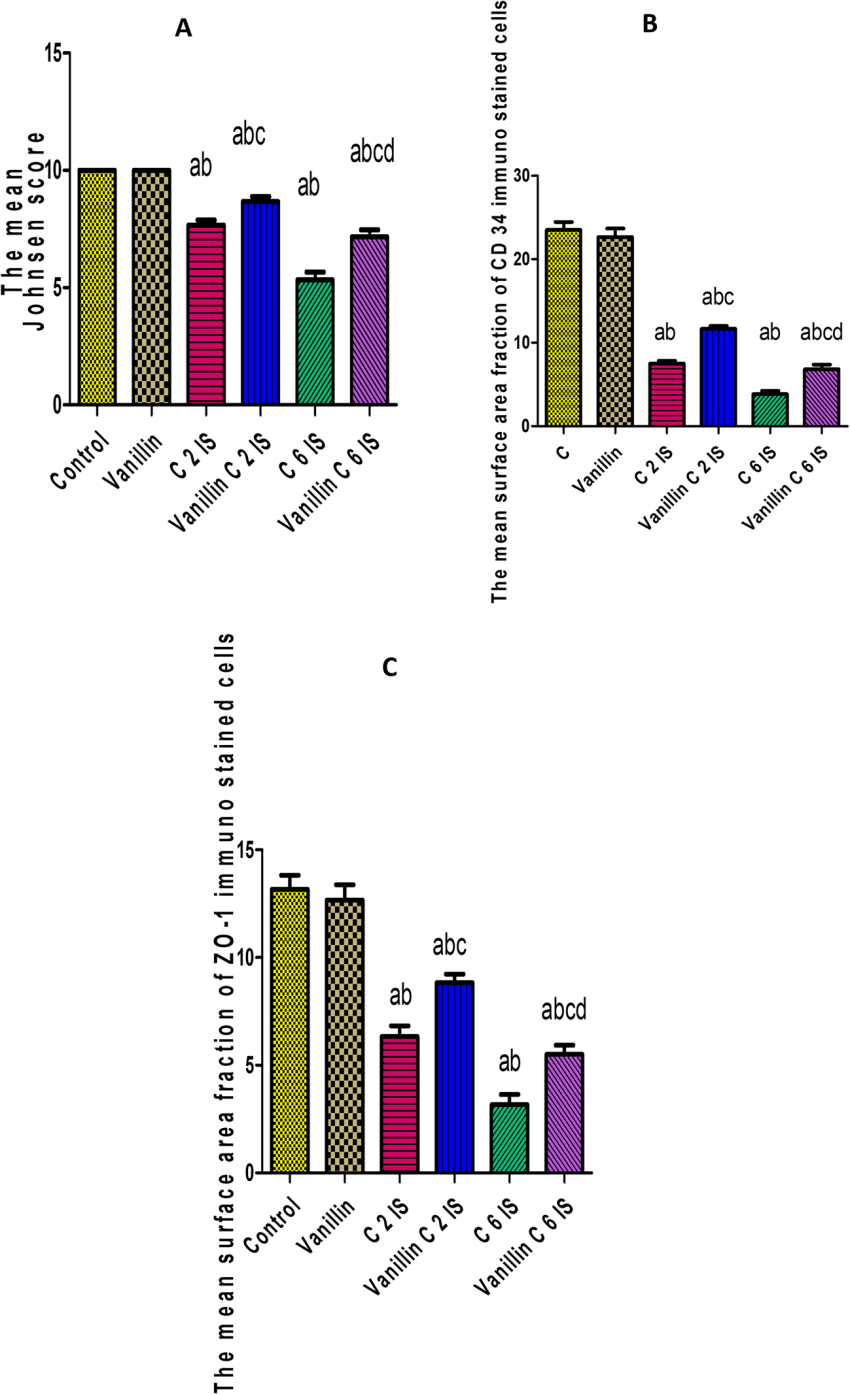
(**A**) The mean Johnsen score. (**B**) The mean area fraction of CD34. (**C**) The mean area fraction of ZO-1. Results represent the mean ± S.E. (**a**) Significant difference from the control group, (**b**) significant difference from the vanillin group, (**c**) significant difference of treated from its corresponding stressed group, (**d**) significant difference of vanillin C 6 IS from vanillin C 2 IS, *P* < 0.05. C 2 IS: chronic 2-h immobilization stress; C 6 IS: chronic 6-h immobilization stress

#### Docking study (in silico analysis)

**Fig. 7:**
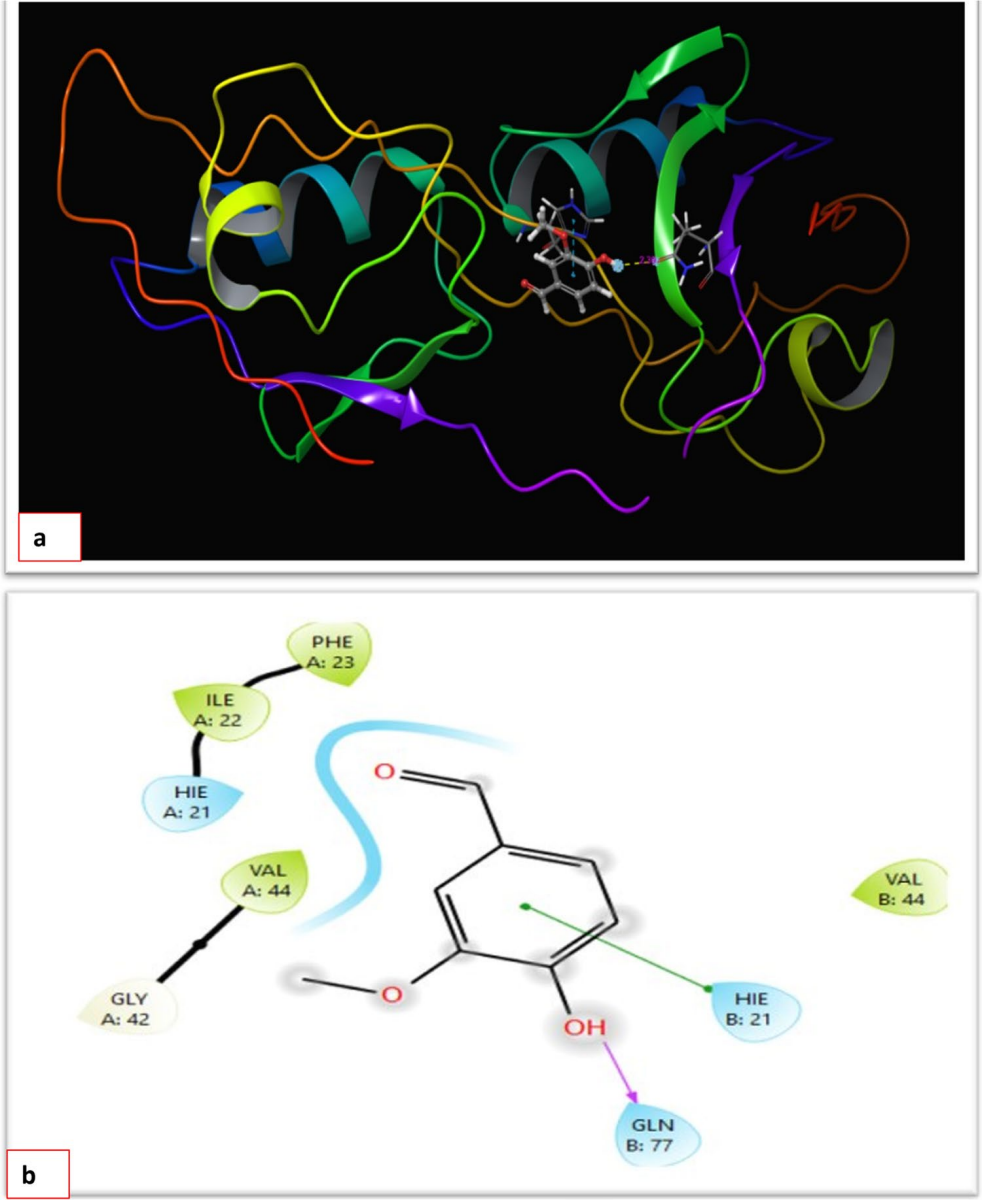
**a** Interaction of 4-hydroxy-3-methoxybenzaldehyde with His 21 and GLN 77 residues in the active site. **b** Interaction of ligand with amino acid residues

In this study, the binding mode of zonula occludens-1 receptor was investigated by doing computational analysis, glide docking. The docking result analysis is described in Fig. 7b. 4-Hydroxy-3-methoxybenzaldehyde showed a good docking score against the receptor. The G-score, H-bond distance, number of hydrogen bonds, interacting residues, and glide or binding energy of each drug molecule are displayed in Table 3. The above parameters determine how stable the drug docking is with the protein; hence, the binding interactions characterize the drug’s level of interaction with the protein zonula occludens-1 receptor that was obtained from the protein data bank using PDB ID: 2JWE. In docking studies involving the target zonula occludens-1 receptor and the ligand, the residues number GLN 77 and His 21are crucial because they act as the active site.

**Table 3:** Schrödinger docking results

Drug name	G-score	Binding energy	No. of H-bonds	Residues	H-bond distance in Ǻ
4-Hydroxy-3-methoxybenzaldehyde	− 4.932	− 19.879	1	GLN 77 His 21	2.39

#### Molecular dynamic simulation

**Fig. 8:**
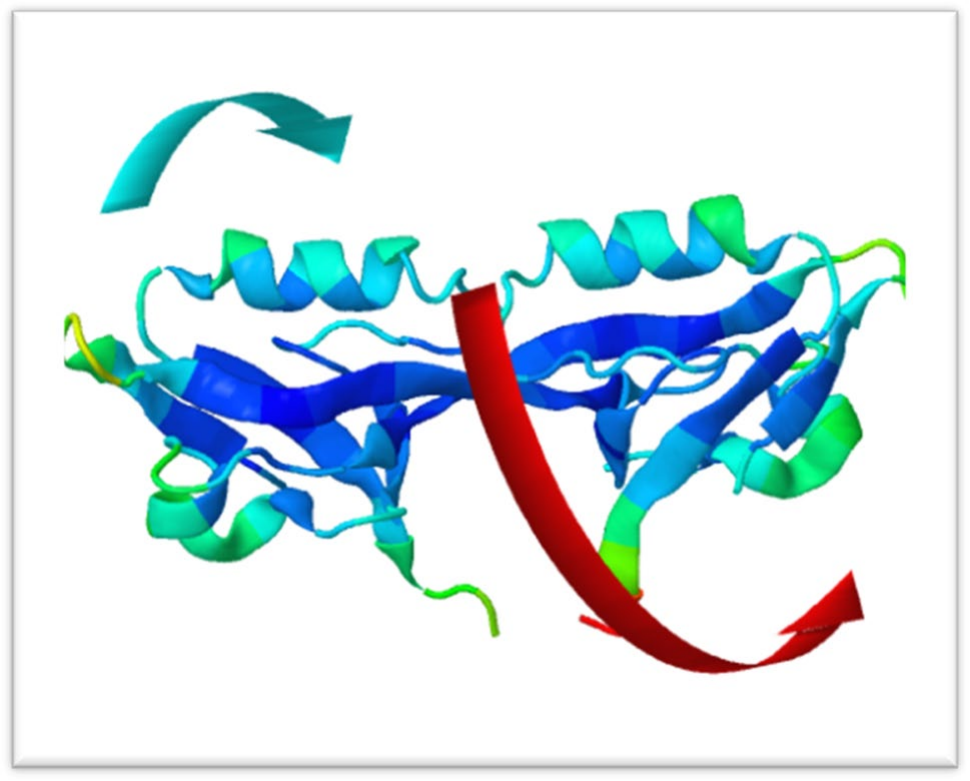
NMA of the docked complex was used to evaluate molecular mobility. (Two colored afine-arrows illustrating the mobility of the domain)

**Fig. 9:**
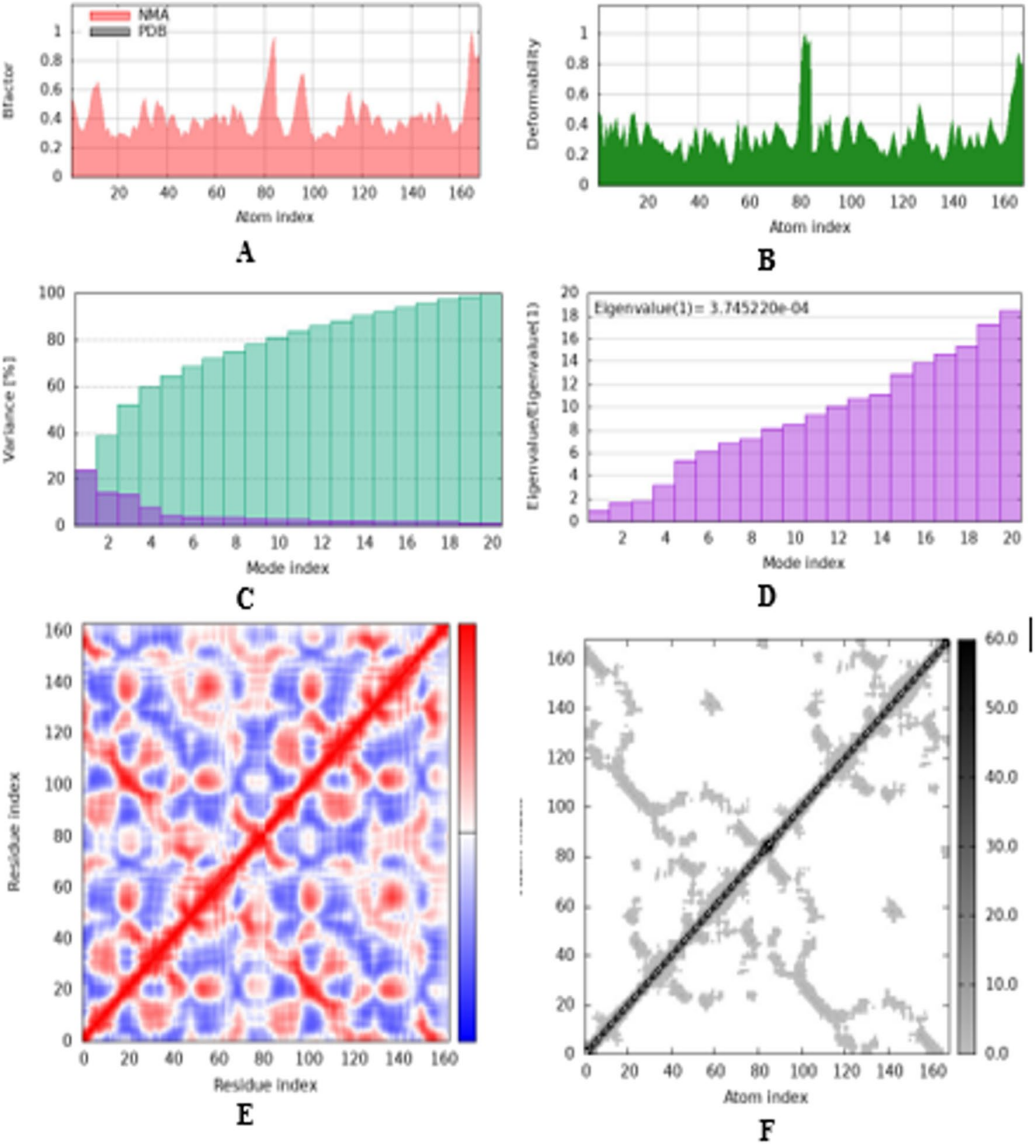
Results of iMODS **A** B-factor; **B** deformability plot; **C** variance plot; **D** eigenvalue; **E** covariance matrix analysis; **F** elastic network model

Molecular dynamics simulation was performed on the docking complex (2JWE-vanillin) using iMOD server (iMODS) (López-Blanco et al. 2014). iMODS was used for the analysis of the structural dynamics of the docking complexes, in addition to determining the molecular motion. iMODs server is a customable and easy to use online server. In addition, this web server provides complicated deformability, variance, covariance map, eigenvalues, B-factor, and elastic network. The docked PDB files function as input files, which are uploaded to the iMODS server. The results were shown with all the parameters left at their default settings. Biological macromolecules must possess flexibility in order to interact with substrates or protein–protein interactions machinery. Consequently, using NMA analysis along with the coordinates of the docked complex, iMODs computes both molecular mobility and structural flexibility. Two colored arrows in Fig. 8 illustrate the domain’s mobility as guided by the server output, with the two clusters indicating the NMA’s mobility. The deformability of the complex is mostly determined by the unique distortion of each residue (Cα atom), which is indicated by colored hinges in the chain (Fig. 9B). The eigenvalue that the server determined was 3.745220e − ^04^, as shown in Fig. 9D. Furthermore, each normal mode has an inverse relationship between the eigenvalue and the variance (Fig. 9C). Although the B-factor plot depicts the stable structure docked molecules displayed in Fig. 9A, the B-factor graph displays the average RMS. The covariance matrix is a color-coded graph that shows correlated, uncorrelated, and anti-correlated motions as, respectively, red, white, and blue (Fig. 9E). Docked protein molecule (Cα) atoms are connected by “springs” of different strengths, as depicted by the darker greys in the elastic network model, which represents the stiffer springs (Fig. 9F) (Desai et al. 2012) (Jayaprakash et al. 2022).

## Discussion

Testicular morphology and function are dependent on appropriate microenvironmental factors such as proper temperature, blood supply, hormone stimulation, and chronic stress exposure (Ribeiro et al. 2018). Stress-induced reproductive damage is more likely due to chronic stress than acute stress. Chronic stress is one of the most important challenges in livestock production; it can cause immune system depression, increased disease sensitivity, and reproductive impairment (Abdel-Fattah and El-Sayed, 2018).

Immobilization stress (IS) is a common stress-research model in experimental animals. It is a combination of physical and mental stress, in which movement is restricted and the animals are separated from all other groups in a confined area. Previous investigations have revealed that separation suppresses the hypothalamic–pituitary–adrenal axis and the hypothalamic-pituitary–testicular axis, and that constraint stress limits male reproductive capacity (Mustafa et al. 2019).

In the current study, chronic IS significantly elevated plasma corticosterone while decreasing plasma testosterone and LH concentrations, which was more pronounced with 6 h of immobilization than with 2 h. Previous research found that male rats exposed to chronic IS exhibited abnormal sexual behavior and lowered fertility. It has been shown that the activation of glucocorticoids inhibits male reproductive functions such as testicular spermatogenesis, steroidogenesis, and epididymal sperm maturation events via suppression of the hypothalamo-pituitary–testicular axis by increased glucocorticoids in rats subjected to restraint (immobilization) stress (Venkaiah et al. 2022).

At the cellular and molecular levels, stress responses can induce oxidative damage by compromising anti-oxidant defenses, as seen in the current study by a significant decrease in testicular catalase concentration; as a result, stress has been connected to the pathophysiology of numerous illnesses (Zhu et al. 2014). Enzymatic anti-oxidants such as catalase act as first line of defense enzymes against the excess generation of superoxide’s and peroxides, respectively, in cellular systems (Venkaiah et al. 2022).

Nuclear factor erythroid 2-related factor 2 (Nrf2) is a redox-responsive transcription factor that prevents oxidative damage by regulating intracellular redox states. It was reported that Nrf2 prevented the formation of oxidative stress during spermatogenesis (Guo et al. 2017). Under oxidative stress conditions, NrF2 dissociates from the Nrf2-Keep1 (Kelch-like ECH associating protein 1) complex and accumulates in the nucleus, regulating cytoprotective genes such as heme oxygenase-1 (HO-1), catalase, glutathione peroxidase (GSH-PX), and glutathione sulfhydryl transferase (GST) (Karna et al. 2020). This was confirmed in the present study by a significant decrease of Nrf2 concentration in stressed groups.

Stress activates the sympatho-adrenal medulla and HPA axis, leading to the secretion of catecholamines and glucocorticoids. These hormones can affect immune cells and cytokine production, including IL-6 and TNF-α. Cytokines serve an important role in bidirectional communication between the neuroendocrine and immune systems. Stress has a variety of effects on immunological function, depending on the type and duration of stress. Stress-induced TNF-α secretion has been observed in various cell types (Saber et al. 2019), including testicular tissue as shown in the current study.

The current work detected a significant structural alteration in the testis after 2 and 6 h of chronic IS; however, these changes were more pronounced in the 6-h stress group in the form of degenerated, irregular, and small-sized seminiferous tubules. This was confirmed by Ribeiro et al. (2018) who suggested that the seminiferous tubule’s diameter was reduced by 6% in the stressed group (286 ± 14.1 μm) if compared with that in the control group (305 ± 8.7 μm).

The current study also showed vacuolated and apoptotic spermatogenic cells, as well as focal thicken basement membranes. In addition, there were damaged Leydig interstitial cells, numerous congested blood vessels, inflammatory cells, and acidophilic chemicals in the interstitial spaces between tubules. Furthermore, this study discovered that unattached germ cells are frequently found in the tubular lumen. This was in accordance with El-Naggar et al. (2020) who stated that chronic stress triggered huge damaging alterations, including deformation of the seminiferous tubules and the death of germ cells. Spermatogenic cells exhibited apoptotic characteristics such as pyknotic and degenerated nuclei, decreased height of the seminiferous epithelium, complete absence of spermatozoa in some tubules, cytoplasmic vacuolations of spermatogenic cells, and detachment of germ cells from the basement membrane in some tubules. They also noted that the interstitial tissue comprised dilated and congested blood vessels, as well as hyalinized acidophilic interstitial material, vacuolation, and degeneration of the interstitial Leydig cells. It was suggested that intraluminal debris attributed the exfoliation of the germ cells to loss their contact with the cytoplasmic processes of the surrounding Sertoli cells (Mohammed et al. 2018). In addition, these changes were attributed to the occurrence of oxidative stress (Elshaari et al. 2012).

In the current study, a wide interstitial with homogeneous acidophilic material was discovered. It may be caused by an increase in vascular permeability (Mohammed et al. 2018).

The current work revealed that the seminiferous tubular Sertoli cells of tissue sections from testis-stressed groups had weak and diffuse ZO-1 immuno-staining. It is possible that chronic stress caused deformation of the actin cytoskeleton of Sertoli cells, which are an integral component of the blood-testis barrier (BTB), explaining the lack of ZO-1 immunological staining. This result is consistent with an earlier report demonstrating that the toxic effect of cadmium chloride exposure can induce fragmentation of actin microfilament bundles in rat seminiferous epithelium and consequently lead to a reduction of actin expression by as much as 50% of the control value by 48–96 h (Wong et al. 2004). This was also confirmed by Kolbasi et al. (2021) who had concluded that in mice, chronic unexpected stress disturbed BTB integrity and sperm parameters. A decrease in ZO-1 expression levels could be postulated as the causal factor.

The discovery of telocytes in a variety of human organs opened up new avenues for research in medicine. Since then, numerous studies on telocytes have been done to find their activities in the human body and to explore potential clinical applications that could aid in the treatment of a variety of disorders. Previous studies suggested that telocytes might contribute to cell signaling, angiogenesis, organ regeneration and repair, apoptosis, and, more recently, the formation of a novel type of tissue barrier. There is proof that telocytes exist at different phases of organ development (Condrat et al. 2021).

Based on the current study “evidence from immune-histochemical studies using telocytes-protein marker CD34,” it could determine the presence of telocytes in the rat testis and how they diminish with immobilization stress. The close interaction of relatively long processes of telocytes with other cell types such as Leydig’s interstitial cells, stromal cells, and blood vessels indicates their likely significance in cell–cell interrelationship and tissue coordination. This was agreed with Abe (2022) who reported that CD34 + PDGFRα + telocytes with long and moniliform telopodes form reticular networks with various cell types as LCs, PMCs, and blood vessels, indicating their potential role both in cell–cell communications and tissue homeostasis.

Vanillic acid is a phenolic derivative of edible plants and fruits that has antimicrobial, ant filarial, and antibacterial properties. It can be found in a wide range of fruits, olives, whole wheat, and cereal grains, as well as wine, beer, and cider. Vanillin’s in vitro antioxidant actions include free radical scavenging, reducing power, and prevention of lipid peroxidation. Furthermore, vanillin decreased lipid peroxidation products and dramatically restored enzymatic and non-enzymatic antioxidants in the plasma of hypertensive rats (Ogunlade et al. 2022).

Vanillin treatment reduced plasma corticosterone and testicular TNF-α levels while increasing plasma testosterone, LH, catalase, and Nrf2 levels. Immobilization stress for 2 h resulted in a greater improvement than 6 h. It might be argued that vanillin treatments reduced the level of testicular tissue inflammation, hence reducing oxidative stress. This was in the same line of Lan et al. (2019) who reported that it has been previously shown that vanillin and its derivatives can protect heart, brain, and liver tissues in ischemia/reperfusion injury models by lowering oxidative stress levels. Vanillin has shown potential anti-oxidant activity as it regulates scavenging of reactive oxygen species with increasing anti-oxidant enzymes as catalase and has also anti-inflammatory action by reducing inflammatory cytokines (interleukin 6 [IL-6], nuclear factor κB [NF-κB], and tumor necrosis factor α [TNF-α]) and oxidative stress (Llatje et al. 2016; Du et al. 2022). Furthermore, the presence of polyphenolics, carotenoids, and other antioxidant elements may account for vanillin’s scavenging actions. This supports previous research on the antioxidant and anti-inflammatory activities of vanillin (Bezerra et al. 2016; Lan et al. 2019). Vanillin suppresses NF-κB activation, COX-2 gene expression, and pro-inflammatory indicators like NO and iNOS (Olatunde et al. 2022).

Additionally, Younis et al. (2020) reported that vanillin administration effectively reduces inflammation by suppressing TNF-α and increasing IL-10 levels. Furthermore, vanillin’s antioxidant action has been linked to increased Nrf2 expression and, as a result, downstream antioxidant molecules. Because of its antioxidant, anti-inflammatory, and anti-apoptotic qualities, vanillin protected against increased testicular apoptosis and fibrosis, as well as enhanced testicular telocyte quantity and regenerating capacity.

The present data clearly demonstrated that administration of vanillin before and with immobilization stress for 2 or 6 h resulted in marked recovery of inflammatory changes and improvement of structural architecture of the testis, with the exception of minimal morphological alterations and remnants of degenerative changes in some seminiferous tubules and interstitial spaces between tubules. Furthermore, the current study demonstrated that the seminiferous tubular Sertoli cells of tissue sections of the testis vanillin-treated stressed groups displayed high ZO-1 immuno-staining as reported by Lan et al. (2019) who reported that the protective effect of vanillin depends on regulation of tight junction (TJ) proteins as ZO-1.

Regarding the docking study, docking poses generated by the docking programs can be directly loaded into Discovery Studio Visualizer. Moreover, results from docking run are summarized in Table 4 and Fig. 7a–f. The mean binding energy of the best conformation was found to be − 4.6 (kcal/mol) and the root mean square deviation (RMSD) value was found to be zero. The interaction study revealed the molecular interaction with A: Ala24, A: Lys30, A: Pro31, A: Arg37, and A: Ile49. Vanillin ligand forms a hydrogen bond acceptor with the amino acid residue A: Arg37.

**Table 4:**
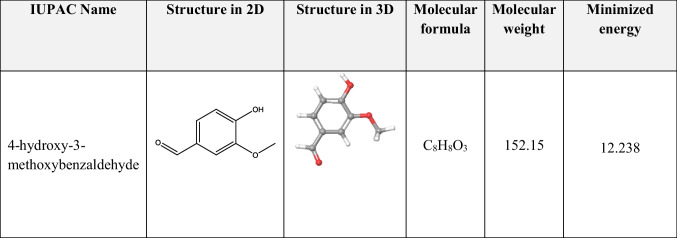
Details of the ligand or drug with the minimized energy

## Conclusion

Taken together, it could be concluded that vanillin protected testicular tissue from the harmful effects of persistent stress. It was considered that the good effects of vanillin were due to its antioxidant and anti-inflammatory properties, in addition to its traditional use as a food additive. This putative testicular protective effect of vanillin highlights the need for further explanation and support from additional investigations. Furthermore, this appears to boost global demand, indicating the need for additional large-scale vanillin manufacturing.

## Data Availability

Availability of data and materials All data is available and would be sent up on request.
